# The Development of Geriatric Assessment and Intervention Guidelines for an Online Geriatric Assessment Tool: A Canadian Modified Delphi Panel Study

**DOI:** 10.3390/curroncol29020073

**Published:** 2022-02-04

**Authors:** Martine Puts, Efthymios Papadopoulos, Sarah Brennenstuhl, Sara Durbano, Nazia Hossain, Brenda Santos, Kristin Cleverley, Shabbir M. H. Alibhai

**Affiliations:** 1 Lawrence S. Bloomberg Faculty of Nursing, University of Toronto, Toronto, ON M5T 1P8, Canada; sarah.brennenstuhl@utoronto.ca (S.B.); brsantos@alumni.uwaterloo.ca (B.S.); k.cleverley@utoronto.ca (K.C.); 2 Department of Medicine, University Health Network, Toronto, ON M5G 2C1, Canada; efthymios.papadopoulos@uhnresearch.ca (E.P.); sara.durbano@uhnresearch.ca (S.D.); nazia.hossain@uhn.ca (N.H.); shabbir.alibhai@uhn.ca (S.M.H.A.); 3 Centre for Addiction and Mental Health, Toronto, ON M6J 1H4, Canada; 4 Department of Medicine, University of Toronto, Toronto, ON M5S 1A8, Canada; 5 Institute for Health Policy, Management and Evaluation, University of Toronto, Toronto, ON M5T 3M6, Canada

**Keywords:** geriatric assessment, Delphi panel study, geriatric oncology, geriatric interventions

## Abstract

Background: There are no guidelines available for what assessment tools to use in a patient’s self-completed online geriatric assessment (GA) with management recommendations. Therefore, we used a modified Delphi approach with Canadian expert clinicians to develop a consensus online GA plus recommendations tool. Methods: The panel consisted of experts in geriatrics, oncology, nursing, and pharmacy. Experts were asked to rate the importance and feasibility of assessments and interventions to be included in an online GA for patients. The items included in the first round were based on guidelines for in-person GA and literature review. The first two rounds were conducted using an online survey. A virtual 2 h meeting was held to discuss the items where no consensus was reached and then voted on in the final round. Results: 34 experts were invited, and 32 agreed to participate. In round 1, there were 85 items; in round 2, 50 items; and in round 3, 25 items. The final tool consists of fall history, assistive device use, weight loss, medication review, need help taking medication, social supports, depressive symptoms, self-reported vision and hearing, and current smoking status and alcohol use. Conclusion: This first multidisciplinary consensus on online GA will benefit research and clinical care for older adults with cancer.

## 1. Introduction

Cancer is a disease that predominantly affects older adults [1]. With the aging of the population, there will be an increase in the number of older adults with cancer [1]. Older adults often have other health conditions that may impact cancer treatment benefits and risks [2]. The American Society for Clinical Oncology (ASCO) and the International Society for Geriatric Oncology (SIOG) have recommended that older adults should receive geriatric assessment to help clinicians and older adults with cancer make treatment decisions [2,3]. Geriatric assessment (GA) involves a multidisciplinary diagnostic process that evaluates an older adult’s medical, psychological, social, and functional capacity, with the aim of developing a plan to address the issues identified in the geriatric assessment and follow-up [4]. A GA can identify health and functional status issues that were previously not known and can impact treatment delivery and treatment outcome [5]. The ASCO guideline [2] recommends that GA includes an assessment of function, comorbidity (including medication review), falls, depression, cognition, and nutrition. The ASCO guideline [2] includes suggestions for assessment tools for these GA domains as well as and recommendations for the clinician to consider when the GA shows an impairment in that geriatric domain [2]. For example, if the assessment tool for the domain functional status shows that the patient has a dependency in multiple basic activities of daily living, recommendations for the clinician could include referral to home care nursing, physical therapy, occupational therapy, etc. [2]. A GA for older adults with cancer has been shown to reduce treatment toxicity [6,7] and improve treatment completion [8], and improves function as measured by the Elderly Functional Index (ELFI) [9]. However, all these GAs were performed in person, and the uptake of GA may be limited due to a lack of geriatric trained teams to conduct GA for all older adults diagnosed with cancer [10,11]. Furthermore, the ASCO guideline, as well as previous conducted Delphi panel studies to develop consensus on GA for older adults with cancer [12,13], included assessment tools that need to be administered in person (such as cognitive screening or performance-based tests of balance, strength, and mobility) and cannot thus easily be transferred to an online environment.

An electronic GA that can be self-administrated by older adults and their caregivers may make GA more accessible to more older adults with cancer. While several electronic GAs have been developed and shown to be feasible [14,15,16,17,18], there are currently no guidelines on what is recommended for an online GA. To facilitate implementation of an electronic GA that includes recommendations for clinicians across Canada, we conducted a modified Delphi panel study. The aim of the Delphi panel was to reach a consensus on an online GA in terms of what assessment tools and what management recommendations (for when the assessment shows a geriatric issue) should be included, with at least one assessment and one recommendation per GA domain.

## 2. Materials and Methods

### 2.1. Study Design

We used a modified Delphi panel approach [19]. The possible assessment tools and recommendations per GA domain were based on a review of current GA guidelines/recommendations for the oncology setting [2,3], previous Delphi panel studies for in-person GA [12,13], and a review of validated assessment tools included in other guidelines. The actual validated assessment tools were included in the online survey so that all participants could review the tool before voting on its importance and feasibility. The GA domains included functional status, mobility/falls, nutrition, mood, social support/situation, medication review, cognition [20], and a miscellaneous category.

In round 1, there were multiple assessment tools and management recommendations listed per GA domain. Experts were asked to rate each assessment tool and recommendation (both called indicator in the Delphi panel method and on which consensus needs to be reached by the Delphi panel) on feasibility and importance. Importance refers to how significant or meaningful the item is to a clinician caring for older adults. Feasibility refers to how feasible the clinician thought it would be to include the item in the online GA tool for patients to self-complete the tool. Feasibility for the recommendations referred to the clinician’s opinion of how feasible it would be for them to implement the recommendation in the care of their patient. The experts were asked to rate for each indicator (i.e., assessment tool or recommendation) importance and feasibility on a 7-point Likert type scale ranging from very important/feasible to not at all important/feasible. Consensus was defined as >70% of the panel scoring ≥6 on importance and feasibility [19,21], and these items were included in the final online GA tool.

Experts were asked prior to starting the survey for round 1 to complete a short survey also in REDCap with 12 questions to obtain demographics and educational and clinical experience in caring for older adults with cancer.

Experts were asked to complete a survey through REDCap for rounds 1 and 2. After each round, items with an importance or feasibility score of <30% or based on feedback by the panel were excluded for the next rounds. As for several geriatric domains such as functional status, we had more than 1 assessment tool included in the first round for voting. If there were comments why certain tools were not appropriate or should be combined, these suggestions were followed (removing assessment tools/recommendations not relevant or new tools to be considered) and brought to the panel in the next round. Before voting in round 3 was undertaken via REDCap, a virtual meeting was held with all experts who were available to discuss the remaining items for which no consensus was reached after 2 rounds to further explore the concerns about feasibility and importance. During this meeting, the wording of some items was adjusted based on the feedback. The experts were asked to vote on these updated items through REDCap during the meeting. The voting in round 3 was limited to experts who participated in the virtual meeting.

### 2.2. Expert Panel

A multidisciplinary expert panel of 34 health professionals in Canada were selected based on their clinical expertise in geriatric medicine, oncology (medical, radiation and surgical oncology, and malignant hematology), nursing, and pharmacy. Experts who were involved in clinical care for older adults with cancer in the past 3 years were eligible to participate. The experts were invited through an email from the 2 leads for this project (MP and SMHA) from across Canada to ensure representation of different experts from different Canadian provinces with slightly different cancer care organizations. Experts received a personalized link to the survey in REDCap, and after they consented to participate in REDCap, they were asked to complete the survey. After voting on each indictor for each GA domain, there was a text box where experts could provide any additional feedback on the domain. A response rate of 70% was required for each round to maintain rigor and 3 email reminders to complete the survey were sent. The survey was live for 4 weeks. While Canada is bilingual, the Delphi Panel was conducted in English as that is the language that all our experts could read and write. Experts received a CAD 25 gift card for their participation in rounds 1 and 2 and a CAD 50 gift card after round 3.

### 2.3. Data Analysis

Sample characteristics were described using frequency counts and percentages for each round. After round 1, the percentage endorsing each indicator was calculated for the importance and feasibility scales separately. Importance/Feasibility was defined as scoring greater than 5 on the 7-point scale. Those that were endorsed for importance OR feasibility were redistributed in round 2 for scoring only on the scale that did not meet the consensus criterion. Indicators that were endorsed by <30% of the sample for importance and feasibility were excluded from the list. Indicators that did not meet the criterion for acceptance or rejection were redistributed in rounds 2 and 3 to be rated on. The comments provided for each indicator were reviewed in conjunction with analysis the quantitative data and additional items were accepted or excluded or reworded based on clinical judgement by SMHA and MP. The same procedure was undertaken after round 2 and round 3, culminating into a final list of accepted indicators.

### 2.4. Ethics

The study was approved by the Ontario Cancer Research Ethics Board and the Health Sciences Research Ethics Board of the University of Toronto. All participants provided written consent online through REDCap prior to the first survey.

## 3. Results

In total, 32 professionals participated in rounds 1 and 2 (94% acceptance rate), and 23 participated in round 3 (72% retention rate). In each of the rounds, approximately 60% of the sample was female, and about 50% of the participants were under 45 years. In the first two rounds, three-quarters were medical doctors (75%), with the other quarter composed of almost all registered nurses (21.9%). In the third round, a slightly lower proportion was medical doctors (69.6%). Medical oncology was the most reported discipline (31%), followed by geriatric medicine (22%) in the first two rounds. In round 3, there was a slightly higher proportion from geriatric medicine (26%) and a lower proportion of medical oncologists (22%). Other disciplines included radiation oncology, geriatric oncology, surgical oncology, haematology, pharmacy, and general surgery. More than half of the participants reported that most of the patients in their clinical practice are aged 70 or older. A quarter of participants indicated that the majority of their patients are frail. Three-quarters indicated that they used geriatric screening tools less than half of their time or rarely. Most participants had either 6–10 or 11–15 years of experience. See Table 1 for the details of the sample characteristics for rounds 1, 2, and 3.

### 3.1. Round One

In round one, 85 items were rated on importance and feasibility (see Appendix A Table A1 for an overview of included assessment tools and management recommendations for each GA domain). The REDCap survey was open between 11 November and 31 December 2020.

Following round 1, 27 indicators were endorsed for importance, and nine were endorsed for feasibility. Out of these, five indicators were endorsed for both importance and feasibility and were automatically accepted (see Table 2 for an overview of all accepted indicators). These five accepted indicators were all about assessment tools: (1) use of mobility devices; (2) use of blister pack/ dosette; (3) living situation; (4) current smoking status; and (5) one-item self-reported hearing difficulty. No items about recommendations were accepted in round 1. Nine indicators were scored <30% for both importance and feasibility and were therefore excluded. Another 28 indicators were removed or modified based on the comments provided (see Appendix A Table A1). In total, 47 indicators were redistributed in round 2 for rating of either importance, feasibility, or both, and three new indicators were added based on the expert feedback provided in round 1. These three items included: assessment of functional status using Instrumental Activities of Daily Living (IADL) with the Older American Resources and Services (OARS) tool, referral of patients with high number of comorbidities (cut-off to be defined) to family physician, internal medicine specialist or geriatrician for assessment and optimization, and assessment of number of alcoholic drinks per week consumed.

Feedback (obtained in the free text boxes included in the REDCap survey after each domain) after round 1 was varied and included: (1) concerns about patient’s ability to complete tools such as the Lawton–Brody IADL tool, whether functional status can be accurately self-reported or not; (2) waiting times for referrals to care in the community affecting the management recommendation’s feasibility; (3) limited resource availability such as pharmacists for medication review affecting the feasibility of this management recommendation; (4) questions about whether the oncologist versus a family doctor was most appropriate to deal with issues identified in the GA such as falls or nutritional status; (5) concerns about the feasibility of cognitive screening tools due to language issues; and (6) no expertise in oncology team members in using cognitive screening tools.

The feedback suggested that the participants answered the items looking at the current resources available in their center, not from the perspective of what would be ideal and should be considered management recommendations to include. Therefore, we added new items in round 2 based on feedback from round 1, and we reworded items and instructions for clinicians with more detailed explanation of importance and feasibility. For feasibility, it was clarified in the survey for round 2 that it meant the feasibility of making the referral, not for the patient to be seen quickly.

### 3.2. Round Two

Round 2 was open between 23 March 2021 and 31 March 2021. Following round 2, four additional indicators were endorsed for importance: (1) assessing the number of falls in previous 6 months; (2) assessing for >10% body weight loss in past 6 months; (3) assessing if the older adults needs help taking medication; and (4) referral to physical therapy (PT)/occupational therapy (OT)/social work (SW) for patients with activities of daily living (ADL) impairment (depending on the specific impairment). One additional indicator for feasibility (referral for more comprehensive fall assessment for those with ≥2 falls in previous 6 months) was also accepted. Out of these 50 indicators, three indicators (assessing the number of falls in the previous 6 months, assessing for >10% body weight loss in the past 6 months, and assessing if the older adult needs help taking medication) were automatically accepted because, between round 1 and round 2, the criterion for consensus had been met. Another four indicators were excluded. In total, 25 indicators were redistributed in round 3 for rating of either importance or feasibility, or both.

The feedback obtained in round 2 included: (1) concerns about patients with lower levels of health literacy, or with limited English language skills and their ability to understand and complete the suggested questionnaires; (2) concerns about feasibility of referrals and wait times in the community for some of these resources; (3) concerns about the length of this online GA tool; and (4) concerns about whether we needed ADL items if IADL items were already included, particularly with concerns over the length of the tool.

### 3.3. Round Three

Round 3 was conducted through a virtual meeting held on 10 June 2021. Prior to the voting in round 3, the leads for this project MP and SMHA provided an update on the previous two rounds. There was a clarification of feasibility and importance rating. Subsequently, the panel went through the 25 items, starting with the ratings of the previous round, discussion of concerns, followed by the voting.

Following round 3 (see Appendix A Table A1), six additional indicators were endorsed for importance: (1) referral to PT/OT/SW for Instrument ADL impairments (IADL) (depending on the specific impairment); (2) recommend measuring postural vitals in patients with ≥ two falls; (3) referral to PT/OT for assessment and management of ≥two falls; (4) asking patients to provide name and address of pharmacy(ies) they use; (5) assessment of depressive symptoms using Patient Health Questionnaire (PHQ)-two items and, if positive, use PHQ-9 items; and (6) assessing the number of alcoholic drinks per week consumed). In addition, 14 items were endorsed for feasibility: (1) recommend measuring postural vitals in patients with ≥2 falls; (2) referral for more comprehensive fall assessment for those with ≥2 falls in previous 6 months; (3) screening every patient for cognitive impairment using Mini-Cog; (4) review of all medications if cognitive impairment is detected; (5) referral to the dietician for those with poor nutritional status; (6) recommendations for nutritional supplements for those with poor nutritional status; (7) assessment of current type and amount of home services; (8) referral to social work to help identify community resources; (9) referral to home care based on MOS-8 score of needing instrumental ADL support; (10) assessment of depressive symptoms using PHQ-2 and, if positive, use PHQ-9; (11) referral to psychosocial oncology for counseling and/or medication if patient has depressive symptoms; (12) counsel patients on benefits of exercise, music, peer support, mindfulness, and sleeping patterns when the patient has depressive symptoms; (13) assessing the number of alcoholic drinks per week; (14) assessment of one-item vision screening.

During the Delphi panel discussion, the following item was added to round 3 as per the experts’ recommendation: “Should a patient be asked how much cannabis they consume?”, so that was included and voted on.

In round 3, three items were automatically accepted on (assessment of depressive symptoms using PHQ-2 and if positive use PHQ-9, recommend measuring postural vitals in patients with ≥two falls, and assessing the number of alcoholic drinks per week consumed). In total, 20 items were accepted in the three rounds; see Table 2 for an overview of assessment tools and recommendations by GA domain that were accepted.

## 4. Discussion

In this modified Delphi study, we aimed to reach a consensus on the assessment tools and management recommendations of an online, self-reported GA. A consensus on assessment tools was reached for seven out of nine domains (mobility/falls, cognitive function, nutrition, depression, medication review, social support, and substance use disorder). No consensus was reached on the inclusion of an assessment tool for functional status and comorbidities. For functional status, the Lawton IADL assessment tool reached a consensus on importance, but feasibility reached only 63%. For management recommendations, a consensus was reached in six out of nine domains. No consensus was reached for management recommendations for the domains functional status, comorbidity, and medication review. For functional status, the recommendation that included referral to physiotherapist, occupational therapist, or social worker for ADL impairments (depending on the specific impairment) reached a consensus on importance, but feasibility only reached 68% endorsement, where 70% was required to reach consensus.

In round 3, the Delphi panel comprised more geriatricians than medical oncologists, and therefore, they may have more experience with these assessments and management recommendations. In this round, many items that were deemed important in rounds 1 and 2 but did not reach a consensus on feasibility reached a consensus in round 3. It is also possible that the discussion that was held virtually about what constituted feasibility (making the referral, irrespective of the availability of services) helped to clarify feasibility better than the additional explanation that was included in the emails and REDCap Delphi panel study. The feedback provided in rounds 1 and 2 clearly indicated that experts had concerns about possible wait times for some of the services that were suggested in the management recommendation items.

In terms of the GA comorbidity, experts raised at the virtual meeting that this should not be included in an online GA; they consider this assessment a key component of their consultation, and thus it has already been performed, and asking patients to undertake this online before the consultation would lead to duplicate questions. In addition, they had concerns about the reliability of patients completing these questions by themselves without any further probing by a clinician, despite the suggested tool being valid and shown to reliably capture common comorbidities [22]. Older adults may also have limited health literacy to be able to answer these questions online, particularly those who are completing the GA in a different language than their native language.

Contrary to our modified Delphi panel, previous Delphi panel studies in the US and Ireland have recommended functional status as an important domain for inclusion for in-person GA for older adults [12,13]. Mohile et al. [12], in the Delphi study in the US, showed that 93% agreed to include both ADL and IADL, only 40% agreed to include only ADL, 80% agreed to include only IADL function, and 93% agreed to include gait speed. In our Delphi panel, we had included measures of IADL and ADL function. However, we did not include this combination of ADL and IADL instruments, as our goal was to identify at least one instrument for each GA domain, and this may explain the discrepancy. O’Donovan et al. [13] in their Delphi panel study also showed inclusion of ADL and IADLs, without recommending particular assessment tools. For the other GA domains, we had not included the same assessment tools, as we selected assessment tools based on a current review of the evidence and what is currently often used in Canada. However, all three Delphi panels recommend assessments of the other domains that were included in all three Delphi studies. A difference between these two published Delphi panel studies and the current Delphi panel study is the inclusion of experts. In the Delphi by Mohile et al. [12] and the Delphi panel of O’ Donovan et al. [13], experts in geriatric oncology were invited to participate. In the study by O’Donovan et al., more than half (55 percent) were members of the International Society of Geriatric Oncology (SIOG) and thus may be more aware of the benefits of GA for older adults with cancer. In our expert panel, we included surgeons, medical and radiation oncologists, geriatricians, and nurses from across Canada to ensure that, when an electronic GA is ready for use, it is relevant and acceptable across Canadian health care settings where older adults with cancer are treated. We purposefully recruited experts who see older adults with cancer but who would not be considered an expert in geriatric oncology to ensure variation on the panel and to ensure the relevance of the assessment for these settings as well. However, it is important to note that these two Delphi panel studies were conducted before the ASCO geriatric oncology guidelines [2] were published. The ASCO guideline includes functional status, and despite having not reached a consensus on feasibility (but borderline with 63% endorsing IADLs and 68% endorsing ADLs), we strongly recommend functional status to be included in any online GA, as it is an important indicator of potential toxicity [2], as well as an important domain for older adults when deciding on cancer treatments, but also it can influence their cognition and ability to remain independent.

Our modified Delphi panel had limitations. Although we had a good initial response rate and good retention of the experts for round 2 of the virtual voting, it was not possible to find a day and time that worked for all our Delphi panel experts for the virtual meeting in round 3 and the response rate for round 3, while above our desired 70% (72%), the balance between oncology experts and geriatric experts tilted towards more geriatric experts, and that may have impacted the results in the sense that more items were accepted for feasibility that were already accepted for importance. A Delphi panel relies on expert opinion, and it is possible that a different panel would have resulted in a different result. We only included experts from Canada, as the study team is developing an online GA for use in Canada. Other Delphi studies on this topic may result in different assessment tools and management recommendations, as these tools and recommendations are health care setting-specific and may thus vary by country depending on different health care systems. However, future studies using online GA using a randomized trial design are needed to show the ultimate benefits of these type of assessments on clinical cancer outcomes. In a next study, we will implement the electronic GA to determine the feasibility and acceptability in daily clinical practice in a variety of settings (e.g., medical oncology, surgical oncology, radiation oncology, academic practice, and community practice).

## 5. Conclusions

The final consensus for an online geriatric assessment tool consists of fall history, assistive device use, weight loss, medication review, need help taking medication, social supports, depressive symptoms, self-reported vision and hearing, and current smoking status and alcohol use. This first multidisciplinary consensus on online geriatric assessment will benefit other researchers interested in developing online geriatric assessment tools.

## Figures and Tables

**Table 1:** Characteristics of expert panel (*n* = 32).

Characteristic	Round 1 and 2 (Same Experts)		Round 3 (Same Experts but Smaller Panel)	
	N	%	N	%
Gender				
Female	19	59.4	14	60.9
Male	13	40.6	9	39.1
Age				
≤45 years	16	50.0	12	52.2
46–55 years	9	28.1	6	26.1
>55 years	7	21.9	5	21.7
Medical Designation				
Medical Doctor	24	75	16	69.6
Registered Nurse	7	21.9	6	26.1
Other	1	3.1	1	4.3
Discipline				
Medical Oncology	10	31.3	5	21.7
Radiation Oncology	4	12.5	2	8.7
Surgical Oncology	3	9.4	3	13.0
Geriatric Medicine	7	21.9	6	26.1
Other	8	25	7	30.4
% Patients 70+				
<25%	1	3.2	1	4.6
26–50%	6	19.4	6	27.3
51–75%	13	41.9	6	27.3
76–100%	11	35.5	9	40.9
Missing	1		1	
% of Patients Considered Frail in Their Practice				
<10%	4	12.9	3	13.6
10–25%	6	19.4	4	18.2
26–50%	13	41.9	8	36.4
>50%	8	25.8	7	31.8
Missing	1		1	
Use Geriatric Screening Tools				
At least half the time	7	21.2	6	27.3
Some of the time or rarely	24	72.7	16	72.7
Missing	1		1	
Clinical Experience with Older Adults				
0–5 years	6	19.4	4	18.2
6–10 years	9	29.0	8	36.4
11–20 years	10	30.3	7	31.8
>20 years	6	18.1	3	13.0
Missing	1		1	
Initial Health Professional Training				
Yes	16	53.3	13	61.9
No	14	46.7	8	38.1
Missing	2		2	
Graduate Training (e.g., PhD, MSc, NP)				
Yes	5	18.2	5	25
No	22	81.5	15	75
Missing	5		3	
Post-Grad Training				
Yes	11	36.7	8	36.4
No	19	63.3	14	63.6
Missing	2		1	

**Table 2:** Accepted indicators in rounds 1, 2, and 3.

Geriatric Assessment Domain	Indicators Accepted for Importance and Feasibility
Functional status assessment tool	None
Functional status management recommendation	None
Comorbidity assessment tool	None
Comorbidity management recommendation	None
Mobility/Falls assessment	Do you use any mobility devices?
	How many falls have you had in the last 6 months?
Mobility/Falls management recommendation	Recommend measurement of postural vitals for someone screens positive for 2 or more falls in the last 6 months (especially if accompanied by dizziness or use of blood pressure lowering drugs)
	Consider a referral for a more detailed falls risk assessment for someone with positive screening for 2 or more falls in the last 6 months
Cognitive function assessment	Screen all older patients for cognitive impairment with the Mini-Cog
Cognitive function management recommendation	Recommend review of medications if cognitive impairment is detected (to reduce medication regime complexity and/or minimize medications that can adversely impact cognition)
Nutrition assessment	Have you lost 10% of your body weight (about 10–15 pounds) or more during the last 6 months?
Nutrition management recommendation	Referral to a dietician (hospital-based or community-based) if a patient demonstrates nutrition risk (as defined by a weight loss of 10% or more in the last 6 months)
Medication review assessment	Do you receive help taking your medication?
	Do you use a blister pack/dosette?
Medication review management recommendation	None
Social support assessments	Patients should be asked their current living situation to assess social support
	Patients should be asked the type and amount of current home services being utilized
Social support management recommendation	Referral for home care be recommended if a patient identifies as needing instrumental ADL support (from the MOS-4 item scale)?
	Referral to social work to help identify community resources and supports if a patient has limited social support
Depression assessment	The PHQ-2 should be used for all older adults and if positive the PHQ-9 be completed
Depression management recommendation	A referral to the family physician for further assessment or a referral to psychosocial oncology (social worker, psychology, psychiatry) for counselling or medication if a patient has depressive symptoms
Substance use disorders and Miscellaneous	How many alcoholic drinks per week do you consume?
	Patients should be asked about their current smoking status
	1-item self-reported hearing difficulty assessment
	1-item self-reported vision difficulty assessment

## Data Availability

The data presented in this study are available in this article.

## Appendix A

**Table A1:** Assessment tools and management recommendations that were rated for importance and feasibility for inclusion in an online GA by the Delphi panel.

Geriatric Domain	Tool Names and Recommendations Included	Round 1 Results % IMPortant/FEASible	Round 2 Results % IMPortant/FEASible	Round 3 Results % IMPortant/FEASible
Functional status assessment tools	Lawton IADL	*IMP 74.2* FEAS 50.0	FEAS 48.4	FEAS 63.3
	OARS IADL *new item based on feedback*	-	IMP 64.5 FEAS 51.6	Excluded *
	Katz ADL	IMP 70.0 FEAS 54.8	IMP 67.7 FEAS 48.8	Excluded *
	Karnofsky Performance status	IMP 40.6 FEAS 50.0	Excluded *	
Functional status management recommendations	Referral to a physiotherapist or other exercise professional for strength and balance training and/or general conditioning with positive screening for poor functional status	IMP 62.5 FEAS 6.3	Excluded *	
	Referral to an occupational therapist for assistive device evaluation when positive screening for poor functional status	*IMP 71.9* FEAS 12.5	Excluded *	
	Referral to an occupational therapist for home safety evaluation with screening for poor functional status	*IMP 71.9* FEAS 18.8	Excluded *	
	Referral to a multidisciplinary program (i.e., geriatric exercise program, falls prevention program, cancer rehab and survivorship) with positive screening for poor functional status	IMP 62.5 FEAS 6.3	Excluded *	
	Referral to a social worker with positive screening for poor functional status	IMP 50.0 FEAS 28.1	Excluded *	
	Referral to PT/OT/SW for IADL impairments (depending the specific impairment), *reworded item based on feedback*	-	IMP 67.7 FEAS 29.0	*IMP 86.4* FEAS 68.2
	Referral to PT/OT/SW for ADL impairment (depending on the specific impairment), *reworded item based on feedback*	-	IMP 86.7 FEAS 40.0	Excluded as KATZ ADL was excluded
Comorbidities assessment	Self-report version of the Charlson Comorbidity Index	IMP 50.0 FEAS 18.8	IMP 43.8 FEAS 34.4	IMP 9.1 FEAS 0.0
Comorbidities management recommendation	Referral for a comprehensive medication review to optimize medications in the context of multiple comorbidities	*IMP 90.6* FEAS 18.8	FEAS 34.4	FEAS 23.8
	Referral of patient with high number of comorbidities (cut-off to be defined) to family physician, internal medicine specialist/geriatrician for assessment and optimization. *New item based on feedback*	-	IMP 51.6 FEAS 15.6	Excluded
Mobility/ falls assessment	Number of falls in the last 12 months	IMP 59.4 *FEAS 81.3*	Excluded *	
	Number of falls in the last 6 months	IMP 66.7 *FEAS 84.4*	IMP 75.0	
	Use of mobility devices	*IMP 81.3* *FEAS 87.5*		
Mobility/ falls management Recommendations	Recommend measuring postural vitals if ≥1 falls, in round 2/3 recommended for ≥2 falls	IMP 68.8 FEAS 50.0	IMP 65.6 FEAS 40.6	*IMP 91.3* FEAS 87.0
	Referral for more comprehensive fall assessment if ≥1 falls, in round 2/3 recommended for ≥2 falls	IMP 62.5 FEAS 15.6	*IMP 71.9* FEAS 9.4	*FEAS 73.9*
	Referral to physiotherapist for mobility and balance if ≥1 falls or poor mobility, in round 2 referral to PT/OT for assessment and management of ≥2 falls	IMP 62.5 FEAS 21.9	IMP 68.8 FEAS 31.3	*IMP 81.2* FEAS 68.2
	Referral to occupational therapist for mobility assist devices if ≥1 falls or poor mobility	IMP 60.0 FEAS 21.9	Excluded *	
	Referral for multidisciplinary falls prevention program for >1 falls or poor mobility	IMP 53.1 FEAS 9.4	Excluded *	
	Referral for home safety evaluation for ≥1 falls or poor mobility	IMP 53.1 FEAS 12.5	Excluded *	
	Referral to a home exercise program for ≥1 falls or poor mobility	IMP 50.0 FEAS 15.6	Excluded *	
	Check and supplement if vitamin D level <50 nmol/L for ≥1 falls or poor mobility.	IMP 31.3 FEAS 56.3	IMP 28.1 FEAS 37.5	Excluded
	Review of medications ≥1 falls or poor mobility	*IMP 78.1* FEAS 37.5	FEAS 31.3	FEAS 61.9
Cognitive impairment assessment	Screen every patient for cognitive impairment with a short validated screening tool (e.g., Mini-Cog)	*IMP 78.1* FEAS 35.5	FEAS 40.6	*FEAS 73.9*
	Screen patients with cognitive complaints with screening tool such as Mini-Cog	IMP43.8 FEAS 32.2	IMP 60.9 FEAS 36.4	Excluded *
	Which cognitive function tool should be used: (1) Blessed Orientation Memory Concentration (BOMC) (2) Mini-Cog (3) Mini Mental State Examination (MMSE) (4) Montreal Cognitive Assessment (MoCA) (5) AD8 Dementia Screening Interview	(1) IMP 34.4 FEAS 32.2 (2) IMP 71.9 FEAS 53.1 (3) IMP 46.9 FEAS 29.0 (4) IMP 40.6 FEAS 18.8 (5) IMP 28.1 FEAS 18.8	All excluded except Mini-Cog *	
Cognitive impairment management recommendations	Assess decision making capacity if CI is detected	IMP 68.8 FEAS 21.9	Excluded *	
	Recommendation to involve proxy in treatment decision-making if CI is detected	IMP 59.4 FEAS 21.9	Excluded *	
	Review all medications if CI is detected	*IMP 81.3* FEAS 46.9	- FEAS 43.8	- *FEAS 82.6*
	Recommend Delirium risk counselling if CI is detected	IMP 59.4 FEAS 31.3	Excluded *	
	Referral to social work if CI is detected	IMP 40.6 FEAS 31.3	Excluded *	
	Referral for more detailed neuropsychological testing including a geriatric clinic or memory clinic if CI is detected	*IMP 75.0* FEAS 16.1	FEAS 18.8	- FEAS 56.5
	Referral for cognitive rehabilitation if CI is detected	IMP 28.1 FEAS 3.1	Excluded	
Nutritional status assessment	5% weight loss in past 6 months	IMP 56,3 FEAS 62.5	IMP 34.4 FEAS 34.4	
	2–5% weight loss or BMI < 20 or reduced muscle mass	IMP 31.3 FEAS 25.8	Excluded *	
	Loss of 10% of body weight past 6 months	IMP 68.8 *FEAS 71.9*	*IMP 75.0*	
	Self-report height and weight to calculate BMI and identify those with BMI < 21	IMP 45.2 FEAS 33.3	IMP 28.1 FEAS 21.9	
	Mini-nutritional assessment	IMP 40.6 FEAS 18.8	IMP 18.8 FEAS 9.4	
Nutritional status management recommendations	Referral to dietician for those with poor nutritional status	*IMP 80.7* FEAS 37.5	FEAS 37.5	- *FEAS 82.6*
	Referral meal delivery program for those with poor nutritional status	IMP 43.8 FEAS 12.5	IMP 28.1 FEAS 25.8	
	Recommendations for nutritional supplements for those with poor nutritional status	IMP 62.5 FEAS 50.0	IMP 59.4 FEAS 45.2	IMP 54.6 *FEAS 78.3*
	Referral to social worker for those with poor nutritional status	IMP 15.6 FEAS 12.5	Excluded	
	Referral to physiotherapist for those with poor nutritional status	IMP 3.2 FEAS 6.3	Excluded	
	Referral to occupational therapist for those with poor nutritional status	IMP 9.4 FEAS 6.3	Excluded	
	Referral cancer rehabilitation program for those with poor nutritional status	IMP 25.8 FEAS 0.0	Excluded	
Medication review assessment	Patients list the names and dosage of prescribed and over the counter medication for medication review, in round 2 changed to patients to list names of all prescribed medications, over the counter and supplements	*IMP 78.1* FEAS 23.3	- FEAS 18.8	Excluded *
	Should patients list the dosage of all prescribed and over the counter medications and supplements? *Reworded item based on feedback*		IMP 56.3 FEAS 9.4	Excluded *
	Do you receive help taking your medication?	*IMP 78.1* FEAS 68.8	- FEAS 81.3	
	Do you use a blister pack/dosette?	*IMP 71.9* *FEAS 71.9*		
	In the past month, have you forgotten to take your medication as prescribed?	IMP 53.1 FEAS 53.1	IMP 50.0 FEAS 59.4	Excluded *
	Patient to list names and address of the pharmacy/ies they use	IMP 68.8 FEAS 43.8	IMP 68.8 FEAS 34.4	*IMP 72.7* FEAS 68.2
	Patient to list number of different medications they take on a regular basis	IMP 37.5 FEAS 28.1	Excluded *	
Medication review management recommendations	Flag potential problematic medications with the Beers list	*IMP 84.4* FEAS 37.5	Excluded *	
	Recommend use of blister pack/dosette if patient indicates they sometimes forget their medication	*IMP 84.4* FEAS 48.4	- FEAS 50.0	- FEAS 42.9
	Recommend blister pack/dosette if patient is on a complex regimen (e.g., 5+ daily medications)	IMP 68.8 FEAS 46.9	IMP 56.3 FEAS 58.1	- FEAS 50.0
	Recommend consultation with a pharmacist when the patient uses a certain number of medications (i.e., 10+)	*IMP 75.0* FEAS 43.8	- FEAS 37.5	- FEAS 68.8
Social support/ circumstances assessment	Should the MOS Social Support Scale 8-items be used to assess social support? In round 2, MOS-4 item was used.	IMP 53.1 FEAS 21.9	IMP 40.6 FEAS 21.9	IMP 33.3 FEAS 38.1
	Do you feel safe at home?	IMP 37.5 FEAS 60.0	IMP 40.6 FEAS 58.1	Excluded *
	Do you have the financial ability to pay all bills?	IMP 56.3 FEAS 59.4	IMP 43.8 FEAS 37.5	Excluded *
	Should marital status be asked to assess social support?	IMP 59.4 *FEAS 78.1*	Excluded *	
	Should patients be asked about their living situation to assess social supports?	*IMP 87.5* *FEAS 84.4*		
	Should the patient be asked the type and amount of current home services	*IMP 75.0* FEAS 65.6	- FEAS 65.6	- *FEAS 80.0*
Social support management recommendations	Referral to social worker to help identify community resources	*IMP 90.6* FEAS 43.8	- FEAS 50.0	- *FEAS 76.2*
	Referral to peer support program	IMP 50.0 FEAS 21.9	Excluded *	
	Referral to community-based cancer support program	IMP 50.0 FEAS 25.0	Excluded *	
	Referral for transportation support based on MOS-8 score of needing instrumental support	*IMP 71.0* FEAS 29.0	- FEAS 18.8	Excluded *
	Referral to home care based on MOS-8 score of needing instrumental ADL support	*IMP 78.1* FEAS 37.5	- FEAS 40.6	- *FEAS 87.5*
Depression assessment	Use PHQ-2 and if positive use PHQ-9	IMP 56.3 FEAS 31.3	IMP 48.4 FEAS 22.5	*IMP 85.7* *FEAS 81.0*
	Distress thermometer	IMP 34.4 FEAS 25.0	Excluded	
	GDS-15	IMP 50.0 FEAS 34.4	IMP 32.3 FEAS 12.9	
Depression recommendations	Referral to psychosocial oncology for counseling and/or medication if patient has depressive symptoms	*IMP 87.1* FEAS 38.7	- FEAS 28.1	- *FEAS 81.0*
	Referral to peer support program if patient has depressive symptoms	IMP 43.8 FEAS 25.0	Excluded *	
	Referral to community-based cancer support program if patient has depressive symptoms	IMP 50.0 FEAS 21.9	Excluded *	
	Counsel patient on benefits of exercise, music, peer support, mindfulness, and sleeping patterns when the patient has depressive symptoms	*IMP 71.9* FEAS 25.0	- FEAS 22.6	- *FEAS 81.0*
Risk prediction	Include VES-13 for risk prediction	IMP 46.7 FEAS 36.7	Excluded *	
	Include G8 for risk prediction	IMP 40.0 FEAS 36.7	Excluded *	
Substance use disorder assessment	The Short Michigan Alcohol Screen Test-Geriatric (SMAST-G)	IMP 18.8 FEAS 15.6	Excluded	
	The Senior Alcohol Misuse Indicator (SAMI)	IMP 12.5 FEAS 6.3	Excluded	
	CAGE-AID screening tool	IMP 31.3 FEAS 25.0	IMP 28.1 FEAS 28.1	Excluded
	Alcohol Use Disorders Identification Test (AUDIT)	IMP 6.3 FEAS 6.3	Excluded	
	Should the patient be asked how many alcoholic drinks per week they consume? *New item based on feedback*		IMP 68.8 FEAS 62.5	*IMP 81.0* *FEAS 81.0*
Miscellaneous	Should patients be asked about their current smoking status?	*IMP 81.3* *FEAS 80.0*		
	Include 1-item self-reported hearing difficulty	*IMP 71.9* *FEAS 71.9*		
	Include 1-item self-reported vision difficulty	*IMP 71.9* FEAS 62.5	- FEAS 54.8	- *FEAS 75.0*
	Should a patient be asked how much cannabis they consume? *New item included by Delphi panel experts*			IMP 69.2 *FEAS 76.9*

Excluded * = based on the combination of a low score, multiple assessment tools in that domain that scored better and clinician feedback, it was decided to remove this item for the next round (even if IMP or FEAS was greater than 30%) or the indictor scored IMP and FES < 30%.
